# Benefits and drawbacks of videoconferencing in eldercare from care workers’ perspectives

**DOI:** 10.1177/20552076241297225

**Published:** 2024-12-05

**Authors:** Ville Mustola, Tomi Oinas, Emilia Leinonen, Sakari Taipale

**Affiliations:** 1 Department of Social Sciences and Philosophy, Centre of Excellence in Research on Ageing and Care (CoE AgeCare), 4168 University of Jyväskylä, Jyväskylä, Finland; 2 Faculty of Social Sciences and Business Studies, Centre of Excellence in Research on Ageing and Care (CoE AgeCare), University of Eastern Finland UEF, Kuopio, Finland

**Keywords:** videoconferencing, care worker, eldercare, personal characteristics, user experience

## Abstract

**Objective:**

The purpose of this study is to examine how care workers’ characteristics are related to the perceived benefits and drawbacks of using videoconferencing in the care of older people. The factors chosen for this study are: age, education, perceived information and communication technology (ICT) support, interest in technology, ICT skills and possible prior experience of using videoconferencing tools.

**Methods:**

Our data source was the second wave (2021) of the University of Jyväskylä survey on elder care work (*N* = 3607), collected from four large trade unions in Finland. We used a multinomial logistic regression to group respondents according to their experiences of technology use and a path analysis to estimate the effects of care workers’ characteristics and prior experiences on the perceived benefits of videoconferencing tools in eldercare work.

**Results:**

We found that the personal characteristics of a care worker are associated with both the use of videoconferencing and its perceived benefits and drawbacks. In addition, we discovered that prior use of videoconferencing tools, especially in direct care work, affects the perceived benefits of them. Those who have used videoconferencing in direct care work perceive them as more beneficial for both their own work and for clients.

**Conclusions:**

Based on these results, we suggest that healthcare and social welfare organizations pay attention to the characteristics of care workers, especially when estimating the necessary amount of ICT support. More ICT support should be targeted to those with a lower level of education and higher age.

## Introduction

Advances in information and communication technology (ICT) have significantly transformed eldercare work, which in the Finnish context covers both social and health care professionals. In the ICT-driven delivery of eldercare services, telehealth has emerged as a promising solution to meet the changing needs of an ageing population and to provide more cost-effective care.^1–4^ As an essential part of telehealth, videoconferencing refers to the use of real-time audio-visual communication devices to facilitate remote interactions between care workers and older people in both social care and health care for older adults with care needs.^5,6^ The increased use of telehealth technologies has been claimed to support older people to live independently, to promote active and assisted living and to improve access to health-related services by changing the dynamics of care delivery.^7,8^ Care workers’ perceptions of their working conditions have changed, with a growing sense of urgency and mounting work-related pressure. These heightened work pressures are also related to an increase in non-direct care responsibilities such as more frequent work-related meetings.^9–16^ At the same time, there has been a notable increase in the adoption of telehealth technologies, a development that has been emphasized due to the demands created by the COVID-19 pandemic.^15,17–22^ Despite the current trend towards the introduction of new telehealth services in social and health care, the use of videoconferencing in eldercare, particularly from the perspective of care workers, has not been thoroughly explored. The current study aims to fill this gap by exploring how care worker related factors are associated with the perceived benefits and drawbacks of videoconferencing in the care of older people.

Prior research shows that the implementation of ICT in healthcare comes not without challenges. It has been shown that in the rapidly changing context of healthcare, approximately half of the ICT projects fail due to financial, social, cultural, managerial and technical issues.^23–25^ Typical causes of such failures include a lack of end user involvement in the implementation process, unclear definitions of the expected benefits of the technology, user perceptions of the technology, possible user resistance and the complexity of the actual care environment.^23,25–28^ On the contrary, user involvement in the development and implementation of these technologies has been shown to be a key success factor.^23,29–33^ All this suggests that both eldercare workers and people receiving care, as end users of videoconferencing tools, have a central role to play in when considering the successful use of videoconferencing tools in the care of older people.

The Technology Acceptance Model (TAM) and its extensions have been widely used to predict individual acceptance and use of technology in healthcare.^34,35^ However, TAMs suit poorly in organizational contexts^36^ where the use of technology is involuntary^37^ and the users’ work environment, cultural background and individual characteristics have not been thoroughly considered.^34,36–40^ In this study, we assume that the use of videoconferencing tools is required by the organization, and we examine how care worker-related factors – namely age, education, perceived ICT support, interest in technology and ICT skills and possible previous experience of using videoconferencing tools – are related to the perceived benefits of videoconferencing in care for older people. The research questions defined for the study are:

RQ1: Which personal characteristics of a care worker are related to the use of videoconferencing?
RQ2: Are there differences in the perceived benefits depending on prior experience of videoconferencing?
RQ3: How are the personal characteristics of a care worker associated with the perceived benefits of videoconferencing use?

Previous research shows that age is strongly associated with ICT skills, usage and attitudes towards new technologies.^38,41–43^ On the one hand, older age has been perceived to relate to lower ICT skills,^44^ and on the other hand, generations born in or after the 1980s have been found to have an advantage in ICT skills.^45^ The literature has also highlighted that education has a significant impact on a person's attitude towards the use of ICT and, consequently, on their willingness to use digital technology – more education means a more positive attitude.^42^ In addition to attitudes, education is related the ability to solve problems in technology-rich environments.^43,46,47^ As technology has become a more fundamental part of modern working life, it also requires workers with a lower level of education, such as vocational training, to engage in innovative forms of problem solving.

Regarding ICT support in the context of eldercare, prior research is limited. Adequate user training and support have been identified as key factors in the successful use of ICT.^48^ Similarly, there is little research on ICT skills and interest in technology in the field of eldercare work. However, these issues have been widely studied, for example, in the field of education research. These studies show that ICT skills, or individuals’ perceptions of their skills, and interest in technology are positively correlated,^49^ which can be explained by the social use of ICT.^50^ There is also a positive relationship between performance expectancy and interest in using technology, and between interest in using technology and the conditions that support its use.^38^

Based on the literature presented above, we created a hypothesized model of factors and their relationships to the perceived benefits of videoconferencing tools. We hypothesize that age is negatively related to education level and interest in technology but positively related to ICT support and the lack of ICT skills. Likewise, we assume that education is positively related to interest in technology but negatively related to perceived ICT support and the lack of ICT skills. We also expect perceived ICT support to have a negative effect on the respondent's lack of ICT skills but a positive effect on the perceived benefits of videoconferencing tools. Furthermore, we expect a lack of ICT skills to be negatively related to interest in technology and the perceived benefits of videoconferencing tools. We hypothesize that both interest in technology and education level are positively related to the perceived benefits of videoconferencing tools.

Recent research has reported several benefits of videoconferencing in eldercare. Videoconferencing can be used to provide remote health care for older adults in care homes through assessment, management of health care, clinical support and diagnostics.^51,52^ In addition, the use of videoconferencing has been found to reduce the social isolation of older people by offering opportunities to connect with family and friends, especially in unconventional conditions such as stay-at-home orders.^53,54^ It can also reduce the inconvenience of travel for clients and thus ensure better continuity of care.^53,55^ From a care worker's perspective, remote technology can distance them from potentially poor working conditions, such as physical and psychological violence.^56^ Previous research also reports some limitations related to the use of videoconferencing in eldercare. There are sometimes limitations in communication cues as well as a limited view or low camera quality,^57^ leading to concerns of not being able to fully identify and understand clients’ medical issues, among others.^58,59^

Existing research does not provide a clear answer to the question of how exactly videoconferencing should be used in care alongside traditional in-person care to benefit clients, staff and administration and to ensure that care meets high ethical standards.^58–61^ For example, in a randomized controlled trial study, a group of patients visited remotely had better blood pressure results than a group patients visited in-person at their homes.^61^ A review of 12 research articles indicated that, generally, both clients and nurses were pleased with the quality of videoconferencing care.^54^ In many previous studies,^57,62^ videoconferencing has been combined with in-person visits instead of replacing them, which might partly explain the high satisfaction with the studied technology. Older people have been shown to prefer videoconferencing over telephone calls but do not think it as good as in-person visits.^63^ This implies that videoconferencing should perhaps not replace in-person visits but be considered as optional or complementary service when in-person visits are not possible.

Finally, in order to understand the context of the study, it is necessary to introduce some specific features of the Finnish eldercare landscape. Finland has a relatively wide age-spatial distribution. This means that many old people live in remote municipalities or in municipalities close to towns or large cities. Simultaneously, the counties responsible are struggling with how to respond to the increasing need for eldercare services with deficit budgets.^64^ The largest occupational groups in the Finnish eldercare system in 2018 were practical nurses and assistive staff, who together made up more than 80% of the eldercare workforce.^65^

In Finland, the use of digital technology has become more common in both home care and residential care over the last years.^64,65^ In 2018, telehealth was used in 40% of home care units in the public sector and in 10% of home care units in the private sector.^66^ The COVID-19 pandemic slightly increased the use of digital tools in Finnish health and social services, including videoconferencing.^19^ It also helped care workers to overcome their reluctance to use telehealth in their work. For example, among home care workers, the use of videoconferencing made their work more meaningful in the context of social distancing rules, as it allowed care workers to maintain and deepen the care relationship with their clients.^67^

## Data and methods

### Data

Our analysis is based on the second wave (2021) of the University of Jyväskylä's panel survey on eldercare work, collected from four large trade unions in Finland. The aim of the nationwide survey was to collect information on the working conditions and use of technology among eldercare workers in Finland. Respondents for the first wave of survey data (*n* = 6903) were collected from the members of four trade unions (SuPer, Tehy, JHL and Talentia) in April 2019. Besides the trade union membership, the inclusion criteria involved that the respondent had to be engaged in ‘daily health care, social care, rehabilitation, or social work with elder or otherwise participate in producing, developing, or managing services for older people’. If not fulfilling these criteria, respondents were excluded from answering the online questionnaire. In the Finnish context, such services are provided by both the social welfare and health care sectors, and eldercare workers can be both social welfare and health care professionals. The number of individuals in the target group varied. For SuPer, the survey was sent using random sampling to every other member of the target group of 38,000. The final sample size was 18,106 respondents with valid email addresses. For Tehy, sampling consisted of two different samples: (1) those among the target group of the survey based on employer information (responses 1,760, sample 7859) and (2) random sampling by including every third member in the member register (responses 666, sample 9600). The sample size was ultimately 17,459. For JHL, the survey was sent to every other member of the target group of 11,000. The final sample size was 4768 respondents with valid email addresses. For Talentia, the members in the potential target group were defined using their titles and education level (8390), all of which were sent the survey. Due to missing or inactive email addresses, the final sample size was 7521 respondents. Of the 6903 responses to the survey, those outside the target group or who declined to answer for other reasons were omitted (528), resulting in the final sample of 6375 responses. Of these, 3607 agreed to participate in further data collection rounds.

The data was collected with an online survey using the 1ka (https://www.1ka.si/d/en) application. The second round of data was collected in April-May 2021 (*n* = 1679). The questionnaire was administered only to the respondents who agreed to participate in the follow-up surveys in the first round. According to drop-out analysis, there was no notable selection of respondents for the second data collection round. The questionnaire did not include any copyright questions. Most of the questions were taken from public surveys, such as the Quality of Work Life (Statistics Finland) and Eurobarometer surveys. Some questions were drawn from previous Nordic and Finnish academic surveys, in a few cases slightly adapted to the purpose of this study. The questionnaire also included items developed specifically for this longitudinal study.

### Measures

Following reviewed literature, we focus our analysis on the relationship between respondent age, education, perceived ICT skills, interest in technology and ICT support to the perceived benefits and drawbacks of videoconferencing tools. Age in years (1) was measured as a continuous variable and (2) education was separated into three levels: (i) primary, (ii) secondary and (iii) tertiary. Perception of one's own ICT skills (3) was measured with the question ‘Does the inadequacy of your own IT or ICT skills slow down the performance of your work tasks?’ with response categories: (i) not at all, (ii) somewhat, (iii) quite a lot and (iv) a lot. Interest in technology (4) was measured with the question ‘How interested you are in technology and its development?’ with response categories: (i) not at all interested, (ii) somewhat interested and (iii) very interested. Organizational ICT support (5) was measured with the question ‘Do you receive support for the use of information technology, information systems, devices or applications related to your work?’ with response categories: (i) way too little, (ii) somewhat too little, (iii) almost enough and (iv) enough. The measures of perceptions on videoconferencing tools (6) include four variables: (i) benefits related to one's own work, (ii) benefits related to clients, (iii) drawbacks related to one's own work and (iv) drawbacks related to clients. The response categories for these variables were (i) not at all, (ii) a little, (iii) somewhat and (iv) a lot. In addition, we use (7) occupation (practical nurses (vocational education), nurses (bachelor level education), other), (8) supervisory status (yes or no), (9) employment sector (public, private, non-governmental organization [NGO]) and (10) workplace type (24 h service housing, home care, institutional care, other) as background variables of work situations for analysis of differences in the experience of using video conference tools.

We construct a three-category variable measuring the experience of using videoconferencing tools in work. The categories were (a) uses videoconferencing tools in direct care work (*n* = 220), (b), by which we mean use where the care worker is in direct contact with the client via videoconferencing tool, uses videoconferencing tools in work but not in direct care (*n* = 778), by which we mean other work uses of the videoconferencing, such as work meetings and (c) does not use videoconferencing tools in work (*n* = 449). In the 2021 survey, respondents who reported using either remote connection devices (e.g., smartphones, tablet computers or home care videophones) or remote care applications (e.g., Zoom, Teams or Skype) were asked whether they used these videoconferencing tools in direct care work. The use of remote connection devices was much more common than the use of remote care equipment (67% and 9%, respectively). In the typology, those who reported using remote connection devices or remote care equipment but not in direct care work were labelled as a ‘semi user’ of videoconferencing tools. This semi-user group mostly consisted of those who used only remote connection devices (92%). By contrast, almost a third of those who used such devices in direct care work reported using both remote connection devices and remote care equipment. Nonetheless, the majority (66%) of this group used only remote connection devices.

### Statistics

First, we use a multinomial logistic regression to model the grouping of respondents with regards to their experience of using video conference tools in their work. The selection analysis was conducted with Stata 16.1. Second, we use a path analysis to estimate the effects of ICT support, ICT skills and interest in technology on the perceived benefits and drawbacks of videoconferencing tools in eldercare. We also estimate the indirect and direct effects of age and education on these perceptions. In addition, we employ multi-group analysis to test differences in the overall model depending on the level of experience with videoconferencing tools. For path analyses, we used Mplus 8.4. For estimation, we used the WLSMV-estimator as all variables except for age are defined on an ordinal level. Our models do not include any latent variables. The polychoric correlations between four items measuring benefits and drawbacks clearly indicated a two-factor model in which measures of benefits and drawbacks load to their individual factor. However, with only two indicators for each latent construct, the model was not identifiable. Thus, we decided to form sum scores for the two dimensions and include them as observed ordinal variables in our model. The Cronbach's alpha for benefits and drawbacks was estimated at 0.69 and 0.72, respectively. We used standardized coefficients, which provide a uniform scale for effects and are thus suitable for our analytical purposes. With the WSMLV-estimator, the direct effects of endogenous variables can be interpreted as a normal regression coefficient (standardized in our case). The WLSMV-estimator estimates a continuous latent variable behind the ordinal observed variables.^68,69^

## Results

**Figure 1.**
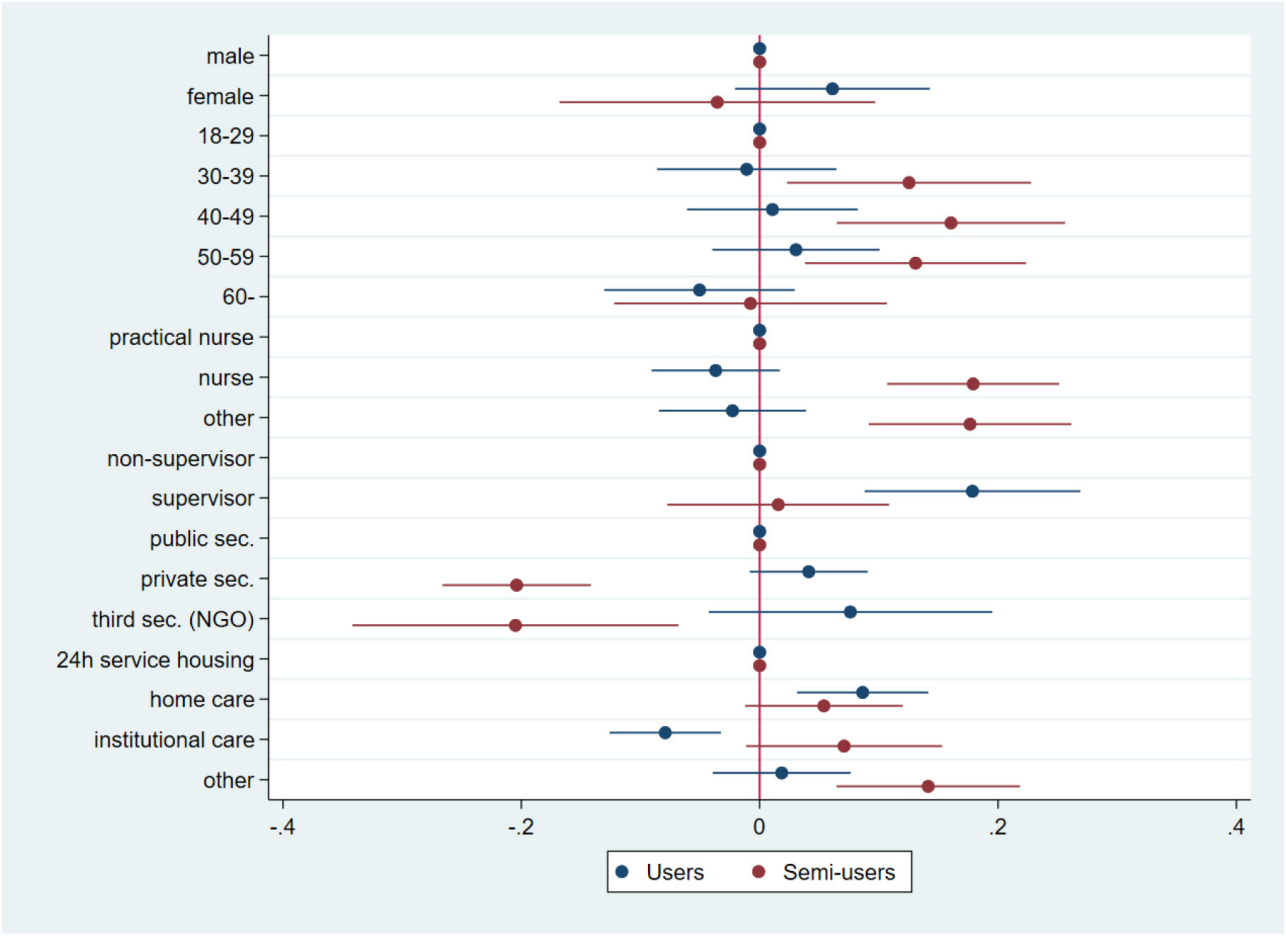
Average marginal effects and 95% CI from the multinomial logistic regression model predicting experience of using videoconferencing tools in care work (*n* = 1373). *Note. User*: uses videoconferencing tools in direct care work; *Semi-user*: uses videoconferencing tools in work, but not in direct care work; *Non-user (ref.)*: does not use videoconferencing tools in work.

Table 1 presents descriptive statistics of the analytical variables by videoconferencing use. The table shows that videoconferencing users and semi-users are typically younger, better educated, work as nurses and are in supervisory positions compared to non-users. Moreover, users are more often employed in the private sector, do home care and are more interested in technology in general. To consider the dependence between these variables, we also examined the grouping of respondents into different videoconferencing user experience groups using multinomial logistic regression. Figure 1 presents average marginal effects^1^ derived from the multinomial logistic model predicting the experience of using videoconferencing tools in care work with different background factors.

**Table 1.** Descriptive statistics (%) of the use of videoconferencing tools.

		Use of videoconferencing tools			*N*	Difference test
		User (*n* = 220)	Semi-user (*n* = 778)	Non-user (*n* = 449)		
Gender	Female	98	97	96	1392	χ^2^(2) = 1.36, *p* = 0.51
	Male	2	3	4	49	
Age	18–29	9	7	14	136	χ^2^(8) =55.91, *p* < 0.001
	30–39	16	18	19	260	
	40–49	28	29	20	380	
	50–59	41	38	31	524	
	60 or more	5	7	16	139	
Education	Primary	1	1	2	17	χ^2^(4) =55.63, *p* < 0.001
	Secondary	68	66	84	1040	
	Tertiary	31	33	14	388	
Occupation	Practical nurse	65	59	83	973	χ^2^(4) =78.92, *p* < 0.001
	Nurse	15	22	11	253	
	Other	20	19	6	220	
Supervisor	No	77	84	96	1256	χ^2^(2) = 56.15, *p* < 0.001
	Yes	23	15	4	187	
Sector	Public	68	81	56	993	χ^2^(4) = 79.13, *p* < 0.001
	Private	28	17	39	349	
	NGO	5	3	5	50	
Workplace	24 h service housing	44	38	62	671	χ^2^(6) = 98.06, *p* < 0.001
	Home care	34	26	16	352	
	Institutional care	5	14	14	182	
	Other	17	21	8	240	
Low ICT skills slow down work tasks	Not at all	40	41	46	613	χ^2^(6) = 6.08, *p* = 0.41
	Somewhat	54	53	47	739	
	Quite a lot	4	5	5	72	
	A lot	2	1	2	19	
Interested in technology	Not at all	8	10	17		χ^2^(4) = 24.01, *p* < 0.001
	Somewhat	60	66	65		
	Very	31	23	18		
Received ICT support	Way too little	23	22	22	305	χ^2^(6) = 12.91, *p* = 0.04
	Somewhat too little	29	32	23	409	
	Almost enough	29	30	36	442	
	Almost enough	19	16	19	243	

Statistically significant differences were found between the three user groups in all factors except gender. Most of these differences occurred between semi-user and non-user groups. Middle aged workers (30–59) had a higher probability of belonging to the semi-user group than the non-user group than the youngest age group. Nurses and those working in other occupations clearly had a higher probability of belonging to the semi-user group than the non-user group when compared to practical nurses. Care workers with subordinates had a higher probability of using video conference tools in direct care work and other work duties than not using them at all. Those working in the private sector or NGOs had a lower probability of being in the semi-user group than the non-user group. There was no statistically significant difference for the user group. Those working in home care were more likely than those working in 24 h service housing to use videoconferencing tools in indirect care work than not using them. In contrast, those working in institutional care had a lower probability than those in 24 h service housing to use videoconferencing tools in indirect care work than not using them at all. In addition, employees in institutional care or other workplaces had a higher probability of being in the semi-user group than the non-user group. Thus, only supervisory status and working in home care (and institutional care, negatively) predicted the use of videoconferencing tools in direct care work.

We also conducted selection analyses separately for both survey years, as a robustness test and to address the role of the COVID-19 pandemic on the use of these devices. The 2019 wave took place before COVID-19, and the 2021 wave collected during COVID-19. Due to data limitations, we were only able to use a binary indicator, that is, whether the respondent used remote connection devices or remote care equipment (the ‘semi-user’ group) in the given year. According to the results (Appendix 1), work and background characteristics predicted similarly the use of videoconferencing tools before and during the COVID-19 pandemic. The most notable difference was that the age of the respondents was not related to the use of videoconferencing tools before the pandemic but did have a statistically significant effect during it (30–59-year-olds used them more often than younger respondents). Thus, there seems to be no difference in the selection between non-user and semi-user groups before and during the pandemic. However, we cannot rule out the possibility of the process of using video conference tools in direct care work changing during our observation period.

**Figure 2.**
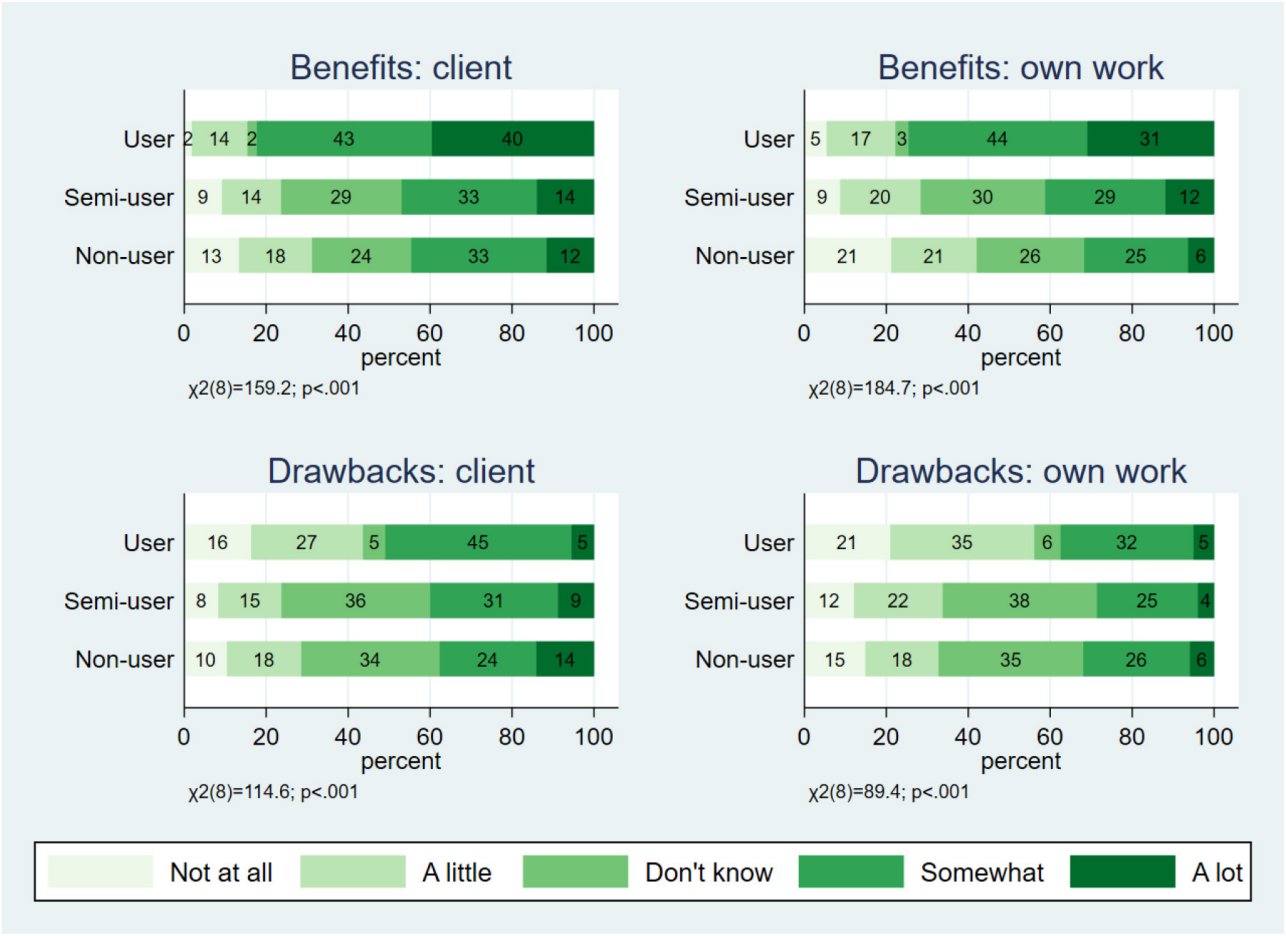
The level of perceived benefits and drawbacks of videoconferencing tools by user experience. *Note. User*: uses videoconferencing tools in direct care work; *Semi-user*: uses videoconferencing tools in work, but not in direct care work; *Non-user*: does not use videoconferencing tools in work.

Next, we examined whether there were differences in the level of perceived benefits and drawbacks of videoconferencing tools based on the respondents’ experiences of using them. According to the results presented in Figure 2, there were statistically significant differences between user groups for all four variables measuring benefits and drawbacks, especially between the semi-user and user groups. Users perceive more benefits in the use of videoconferencing tools – both for clients and their own work – than semi-users and non-users. Users also perceived more drawbacks, both for clients and their own work, than semi-users and non-users.

**Figure 3.**
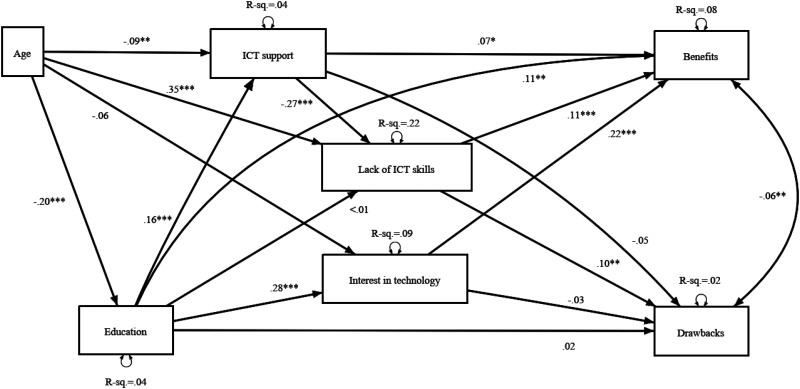
Results (standardized coefficients) from path analysis (WLSMV) predicting the perceived benefits and drawbacks of videoconferencing tools in eldercare work (RMSEA = .040, CFI = .99, TLI = .91, SRMR = .013). *n* = 1576; * *p* < 0.05; ** *p* < 0.01; *** *p* < 0.00.

In the next phase, we conducted a path analysis for the overall sample predicting perceptions of the benefits and drawbacks of using videoconferencing tools in direct care work. As Figure 3 and the associated statistics show, the hypothesized model fits the data well, although some of the expected effects were not statistically significant.

Age had a negative effect on education and a positive effect on lack of ICT skills. Thus, older respondents, on average, had a lower level of education and ICT skills than younger ones. In addition, age had a moderate negative effect on perceived ICT support, contrary to what was expected. Older respondents might need more support with ICT than younger ones and thus, the current level of support was perceived as inadequate. The negative effect on interest in technology was not statistically significant.

Figure 3 also shows that education level had a positive effect on interest in technology and perceived ICT support. The direction of this effect was that more support would be provided for those with a lower level of education. The reasons for this could be explained similarly to the effect found for age: the respondents with lower education levels might require more support than they receive to feel that it is adequate. The perceived ICT support, in turn, had a negative effect on respondents’ lack of ICT skills. The effect of support on the perceived benefits of videoconferencing tools was small but statistically significant. Similarly, there was a small negative statistically non-significant effect on the perceived drawbacks of videoconferencing tools. Lack of ICT skills had a positive effect on both the perceived benefits and drawbacks of videoconferencing tools after controlling for all other variables. Thus, the lower the level of ICT skills, the more benefits and drawbacks the respondents perceived in using videoconferencing tools. Additionally, interest in technology had a positive effect on the perceived benefits of videoconferencing tools, that is, the more interested the respondents were in technology overall, the more benefits they ascribe to the use of videoconferencing tools. In addition, education level had a positive effect on the perceived benefits of videoconferencing tools. A small but statistically significant negative correlation was found between the perceived benefits and drawbacks of videoconferencing tools.

In addition to the analysis presented in Figure 3, we estimated the indirect effects of age, education and ICT support through multiple mediators on perceived benefits and drawbacks. There were statistically significant total indirect effects of education (Beta = .07, *p < *.001) and ICT support (Beta = −.03, *p = *.001) on benefits. Both coefficients were smaller than the direct effects shown in Figure 3. Almost all of the indirect effect of education comes from its effect via interest in technology (Beta = .06, *p *<* *.001). ICT support had only one mediator, namely lack of ICT skills. For age, the total indirect effect was not statistically significant. Age (Beta = .05, *p *<* *.001) and ICT support (Beta = −.03, *p = *.003) had statistically significant total indirect effects on perceived drawbacks. For ICT support, the indirect coefficient was smaller than the direct effect. Most of the indirect effect of age comes from its effect via lack of ICT skills (Beta = .04, *p = *.002). Education did not have a statistically significant total indirect effect on perceived drawbacks. There were also some more complex and statistically significant mediations, but their coefficients were very small.

In the next phase, we estimated a multi-group path model using respondent experience of videoconferencing tools as grouping factor. The three groups were: those who use videoconferencing tools in direct care work (‘users’, *n* = 219), those who use videoconferencing tools in work, but not in direct care work (‘semi-users’, *n* = 774) and those who do not use videoconferencing tools in work (‘non-users’, *n* = 446). We used a Wald test to test for group differences in coefficients. With multi-group analysis, the aim is to estimate whether the hypothesized model differs between those who have actual experience of videoconferencing tools and those who do not. Thus, we examine the difference between expectations and experiences regarding videoconferencing tools.

As shown in Appendix 2, there were some differences in coefficients between these three groups. First, interest in technology had a statistically significant negative effect on perceived drawbacks only for those who have actual experience of using these tools in direct care work (users). By contrast, interest in technology had a statistically significant positive effect on perceived benefits only for those who have some experience of using video conference tools in their work (semi-users). Similarly, education had positive associations with interest in technology and ICT support for those who have some experience of using videoconference tools in their work (semi-users). In other words, there were clear differences in how organization-level and individual-level characteristics were related to the actual versus expected benefits or drawbacks of videoconferencing tools.

## Discussion

### Key findings

Firstly, the results confirm that older respondents, on average, have a lower education level and lower ICT skills.^38,41–44^ They also perceive current ICT support to be inadequate. The respondents with higher education levels were, on average, more interested in technology and were more satisfied with current ICT support,^42^ as also found in previous research. The respondents with more interest in technology and higher education levels perceived more benefits in using videoconferencing tools in eldercare, and this is also in line with previous research.^38,42,49^ Unexpectedly, a lack of ICT skills had a positive effect on both perceived benefits and drawbacks of videoconferencing tools after controlling for all other variables. The results also show the indirect effects of age, education and ICT support through multiple mediators. Hence, the effects of age and education on the perception of video conference tools are apparently partly mediated by ICT support, the lack of ICT skills and interest in technology.

Second, the group differences between users, semi-users and non-users of videoconferencing tools in eldercare indicate that there are some differences in coefficients between the three groups. For example, interest in technology has a significant positive effect on perceived benefits for those who had used video conference tools in their work at least to some extent. It is then clear that the actual experience of using technology in a work context is significant when considering users’ perceptions of the benefits of technology.

Third, the results point out that eldercare workers who use videoconferencing in direct care work see it as much more beneficial, both for their own work and for clients, than those who use it in other types of work. In practice, this means that the use of videoconferencing tools is seen as much more useful for contacting clients as in, for example, online work meetings or medical training for eldercare staff. This outcome is associated with previous findings on the topic of eldercare workers’ perceptions of their work conditions. Eldercare workers’ feelings of increased workload, constant time pressure and lack of possibility to complete their work properly are widely recognized,^9–11^ as are the increases in time used for work-related staff meetings^12^ and other duties that are not actual care work.^13^ Our findings indicate that eldercare workers recognize the value of videoconferencing especially when it can be used for the core function of care work.

### Theoretical implications

As, in our research setting, the use of videoconferencing in eldercare was decided at the organizational level, it was not relevant to examine the acceptance of using the TAM and its extensions.^24–26^ The use of the original TAM model within health informatics has proven to be difficult, and often the model has been extended to fit the dynamic field of health and social services by integrating components and variables in specific contextual settings.^34–38^ This applied to our study as well. We selected users’ perceptions of the benefits of videoconferencing technologies as a core component of our theoretical model because, in an environment where the use of technology has already been decided by the organization, it is central for functional care work.^16^ Based on our results, the tasks which videoconferencing tools are used for are highly related to their perceived benefits. Thus, our study demonstrates that it is not enough to include only technology user, cultural context and a certain technology in the theoretical model, as also noted in previous studies,^36,37,39,40^ but also how and for what purpose technology is used in organizations should be considered.

Our study shows that several issues still require further investigation. First, our analysis which grouped respondents into different videoconferencing user experience groups revealed that supervisory status predicts the use of videoconferencing tools in direct care work. However, we can only hypothesize the reasons behind this finding. For example, those with subordinates might have more control over their work and have wider care work experience. However, future studies should look to explain why supervisory status leads to a higher probability of belonging to the ‘user’ group.

### Practical implications

Our analysis provides several practical implications, especially for organizations considering implementing, or already using, videoconferencing tools in eldercare. First, it is notable that actual experience of videoconferencing tools in direct care work correlates with a more positive perception of this technology related to one's own work but with a more negative perception related to clients. In this regard, it might be beneficial for healthcare and social welfare organizations to provide short-term trial periods for care workers to test videoconferencing tools in direct care work situations before making the decision to implement these technologies. It is worth noting that this study only offers information from a care worker's perspective, but complementary data is required to investigate the client perspective. Based on our data, we also concluded that more ICT support should probably be targeted to those with a lower level of education and higher age. Based on our path analysis for the overall sample, eldercare workers seemingly did not feel that the ICT support provided was adequate. Therefore, further ICT support could increase care worker confidence before initiating a video session.

Our study revealed that those with subordinates are more often users of videoconferencing tools in direct care work and they see the use of this technology in a more positive way. We assume that this could be because they have more influence over their own work. Thus, they can use videoconferencing tools in more flexible and individual ways than those who work as subordinates. Alternatively, those in managerial positions may have a higher level of education, which has a positive effect on interest in technology. Previous literature detailed that, in eldercare, one of the biggest reasons for moral stress is the lack of influence over one's own work^14^ and therefore, we assume that voluntary use of technology may lead to more beneficial perceptions of it. Considering clients’ individual needs and wishes is concordant with care worker ethical codes^16,60,61^ and therefore, it could be desirable to choose either in-place care or care through videoconferencing on occasion. Furthermore, future studies should examine how flexibility and individuality when using videoconferencing tools in eldercare affects the perceived benefits of these technologies.

### Limitations

As with other empirical studies, our study also has limitations. Firstly, we collected data only in Finland (where we had no access to non-union members) and our results do not apply to all social and cultural conditions and further studies are required to address other cultural and societal contexts. Secondly, given the extremely high proportion of females in the target population, we cannot adequately address gender effects. Thirdly, Care for older people is divided into institutional care and home care and their variations. These care settings are diverse and the use of videoconferencing tools in direct care is not as relevant in institutional care, where distance is not a barrier, as it may be in home care. Fourthly, as precise data on the degree of unionization of eldercare workers in Finland are not available, any generalization of these results to all Finnish eldercare workers would be speculative. Future research needs to consider the varying institutional settings when investigating videoconferencing tools in care work.

## Conclusions

Our study provides information on how eldercare workers perceive the benefits and drawbacks of videoconferencing tools in their work. Considering RQ1 and RQ3, we found that the personal characteristics of care workers are associated with both the use of videoconferencing tools and the perception of the benefits and drawbacks of them. Regarding RQ2, we discovered that prior use of videoconferencing tools, especially in direct care work, affects the perceived benefits of them. Based on these results, we suggest that healthcare and social welfare organizations pay attention to the characteristics of care workers, especially when estimating the necessary amount of ICT support. Organizations should also realize that care workers’ concrete experiences of using videoconferencing tools in direct care work increase their perception of both the benefits and drawbacks of these tools.

## Notes

1. Average Marginal Effects (AME) are interpreted as the average change in probability of an event by one unit increase in the predictor variable, keeping everything else constant. AMEs for the omitted reference category of a predictor variable is always zero.

## Appendix 1.

Average marginal effects (and 95% CI) from the binary logistic regression model predicting the use videoconferencing tools in work by survey year (2019: *n* = 8434, 2021: *n* = 1392).

## Appendix 2.

Multi-group path analysis (WLSMV) predicting the perceived benefits and drawbacks of videoconferencing tools in elder care work.

**User**: uses videoconferencing tools in direct care work (*n* = 219)

**Semi-user**: uses videoconferencing tools in work, but not in direct care work (*n* = 778)

**Non-user**: does not use videoconferencing tools in work (*n* = 449)

χ^2^(33) = 41.11, *p* = 0.16, RMSEA = 0.023.
